# SIRT1-Dependent Upregulation of BDNF in Human Microglia Challenged with Aβ: An Early but Transient Response Rescued by Melatonin

**DOI:** 10.3390/biomedicines9050466

**Published:** 2021-04-24

**Authors:** Grazia Ilaria Caruso, Simona Federica Spampinato, Giuseppe Costantino, Sara Merlo, Maria Angela Sortino

**Affiliations:** Department of Biomedical and Biotechnological Sciences, Section of Pharmacology, University of Catania, Via Santa Sofia 97, 95123 Catania, Italy; grazia.caruso@outlook.it (G.I.C.); simona_spampinato@hotmail.com (S.F.S.); giuseppe.costantino@unifg.it (G.C.)

**Keywords:** Alzheimer’s disease, HMC3 human microglia, inflammation, microglial switch, NF-kB, Silent Information Regulator 2 homolog 1, brain-derived neurotrophic factor

## Abstract

Microglia represent a first-line defense in the brain. However, in pathological conditions such as Alzheimer’s disease (AD), a pro-inflammatory switch may occur, leading to loss of protective functions. Using the human microglial cell line HMC3, we showed that exposure to low concentrations of β-amyloid peptide 1-42 (Aβ42; 0.2 μM) initially (6 h) upregulated anti-inflammatory markers interleukin (IL)-4, IL-13, and brain-derived neurotrophic factor (BDNF). BDNF increase was prevented by selective inhibition of SIRT1 with EX527 (2 μM). Accordingly, these early effects were accompanied by a significant Aβ42-induced increase of SIRT1 expression, nuclear localization, and activity. SIRT1 modulation involved adenosine monophosphate-regulated kinase (AMPK), which was promptly (30 min) phosphorylated by Aβ42, while the AMPK inhibitor BML-275 (2 μM) attenuated Aβ42-induced SIRT1 increase. Initially observed microglial responses appeared transient, as microglial features changed when exposure to Aβ42 was prolonged (0.2 μM for 72 h). While SIRT1 and BDNF levels were reduced, the expression of inflammatory markers IL-1β and tumor necrosis factor (TNF)-α increased. This coincided with a rise in NF-kB nuclear localization. The effects of melatonin (1 μM) on prolonged microglial exposure to Aβ42 were analyzed for their protective potential. Melatonin was able to prolong SIRT1 and BDNF upregulation, as well as to prevent NF-kB nuclear translocation and acetylation. These effects were sensitive to the melatonin receptor antagonist, luzindole (25 μM). In conclusion, our data define an early microglial defensive response to Aβ42, featuring SIRT1-mediated BDNF upregulation that can be exogenously modulated by melatonin, thus identifying an important target for neuroprotection.

## 1. Introduction

Alzheimer’s disease (AD) is a progressive neurodegenerative disorder affecting primarily the elderly. A salient feature of AD is that it develops slowly over the years, remaining asymptomatic for up to two decades before diagnosis is possible [1,2]. By this time, neurodegeneration is so advanced that chances for treatment are reduced, accounting at least in part for current failure to develop effective disease-modifying therapies [1,3].

From a molecular point of view, hallmarks of AD are the increased brain levels of the beta amyloid peptide (Aβ) and phosphorylated tau protein, which respectively aggregate into extracellular plaques and intracellular tangles [4,5,6,7]. According to the amyloid cascade hypothesis, initial accumulation of the aggregation-prone 42 amino acid-long isoform of Aβ (Aβ42) is the result of an imbalance between its production and/or clearance, leading to abnormally high concentrations of oligomers that hold potential for neurotoxicity upon chronic exposure [5]. Aβ can directly interact with neuronal surface molecules, damage the cell membrane, and be internalized with ensuing oxidative stress [8]. Interestingly, however, glial cells can respond to rising concentrations of Aβ oligomers activating to oppose its buildup and its neurotoxicity. Microglia, in particular, interact with Aβ through a variety of receptors and are the main effectors of its clearance, exerting an initial anti-inflammatory response [9,10,11,12]. However, the clearing and neuroprotective functions of microglia may become insufficient upon excessive Aβ buildup, triggering a pro-inflammatory phenotypic switch [13]. In agreement, data from both AD patients and animal studies reported an increased expression of neuroinflammatory cytokines with disease progression, which coincided with a significant reduction of BDNF levels in cognition-related brain structures and in serum [14]. On these bases, targeting microglia to enhance/prolong their beneficial functions and halt/delay pro-inflammatory polarization has been proposed to represent a successful strategy [15,16].

Among candidate effectors for neuroprotection against neurodegenerative diseases, including AD, is Silent Information Regulator 2 homolog 1 (SIRT1) [17,18,19,20]. SIRT1 is an NAD^+^-dependent deacetylase that modulates gene expression by deacetylation of histones and transcription factors. Among its targets is NF-kB, accounting for the anti-inflammatory actions of the enzyme [21,22]. In particular, SIRT1 has been shown to affect several processes in the pathogenesis of AD, from Aβ synthesis to tau toxicity, and declines in its levels have been suggested to mirror disease progression [23,24,25].

An interesting candidate activator of SIRT1 is melatonin, an endogenous neurohormone shown to be pleiotropic and neuroprotective in neurodegenerative conditions [26] including AD [27], Parkinson’s disease [28], hypoxia/ischemia [29], and spinal cord injury [30]. Animal and human studies showed that the use of melatonin is safe in short- and long-term treatments. Only mild and no serious adverse effects have in fact been reported so far [31]. Melatonin is able to exert neuroprotection through different cellular mechanisms, including activation of antiapoptotic pathways, upregulation of anti-oxidant enzymes, and inhibition of pro-inflammatory signaling [26,32]. The hormone mainly acts through cell membrane G protein-coupled receptors, MT1 and MT2 [33,34], both widely distributed in different brain areas and expressed by both neuronal and glial cells [35]. In addition, intracellular binding sites have been reported, namely the quinone reductase enzyme MT3 [36] and the retinoic acid-related orphan receptors RORs [37]. Non-receptor-mediated actions reported for melatonin include the direct detoxification of reactive oxygen and nitrogen species [38]. In AD, melatonin-mediated neuroprotective mechanisms include anti-amyloidogenic actions [39,40], synaptic stabilization [41], and promotion of neurogenesis [42]. Clinical studies are currently underway to determine the potential of melatonin administration against sleep alterations and related decline in cognitive functions in AD, with so far positive results [43].

Based on these premises, and moving from our previous work showing the early contribution of microglia to neuroprotection [11], we here aimed to characterize the time course of beneficial microglial responses to low concentrations of Aβ, using an in vitro system to mimic the very initial events in AD development. For this purpose, we used the human microglial cell line HMC3. Furthermore, we evaluated the involvement of SIRT1 and the ability of melatonin to target SIRT1 in order to enhance microglial anti-inflammatory functions, hindering the pro-inflammatory switch.

## 2. Materials and Methods

### 2.1. Drugs and Reagents

Amyloid β peptide 1-42 (Aβ42) from Innovagen (Lund, Sweden) was prepared according to the protocol previously used in our lab [44]. Briefly, Aβ was dissolved in dimethylsulfoxide (DMSO; Sigma-Merck, Darmstadt, Germany) as a 5 mM stock, subsequently diluted to 100 μM in a culture medium, and enriched in oligomers by aggregation at RT for 24 h, followed by at least two freeze–thaw cycles prior to use. Melatonin, EX527 (Santa Cruz Biotechnologies, Santa Cruz, CA, USA), and BML-275 (Enzo Life Sciences Inc., Farmingdale, NY, USA) were dissolved in DMSO as 10 mM stocks and further diluted in a culture medium for experiments. Both EX527 and BML-275 were always added 15 min before other drugs. Luzindole (Tocris, Bristol, UK) was dissolved in DMSO as 50 mM stock and further diluted in culture medium for experiments, where it was always added 30 min before other drugs. Golgi inhibitor brefeldin-A (Thermofisher Scientific, Waltham, MA, USA) was dissolved in DMSO as a 10 mg/mL stock and added during the last 3 h of treatment.

### 2.2. Cell Cultures

The HMC3 human microglial cell line (ATTC, LGC Standards, Manassas, VA, USA) was grown in Eagle’s Minimum Essential Medium (EMEM; Thermofisher Scientific, Waltham, MA, USA) supplemented with 10% fetal bovine serum (FBS; Thermofisher Scientific, Waltham, MA, USA) and penicillin (100 U/mL)/streptomycin (100 μg/mL) at 37 °C and in a 5% CO_2_ atmosphere. Based on experimental needs, cells were plated with the following densities: 800 k cells/well in six-well plates, 15 k cells/well in 96-well plates (all plastic from Falcon, Milan, Italy), or 8 k cells/well in eight-well microslides (Ibidi, Gräfelfing, Germany). For morphological observation, cells were stained with the fluorescent dye FM^®^ 1–43 (5 μM for 15 min; Thermofisher Scientific, Waltham, MA, USA).

### 2.3. Quantitative Real-Time Polymerase Chain Reaction

Cells were collected and total RNA extracted using the RNeasy Plus Mini Kit (Qiagen, Milan, Italy). RNA concentration was determined using Nanodrop spectrophotometer ND-1000 (Thermofisher Scientific, Waltham, MA, USA), and 2 μg of RNA were reverse transcribed using Superscript-VILO kit (Thermofisher Scientific, Waltham, MA, USA) according to the manufacturer’s instructions. Quantitative real-time PCR (qRT-PCR) was performed on a 1:300 dilution of the reverse transcription reaction per sample, using the Rotor-Gene Q and Qiagen QuantiNova SYBR Green Real Time-PCR Kit. Primers are listed in Table 1 and were all from Qiagen. RPLP0 was used as the endogenous control. Melting curve analysis confirmed the specificity of the amplified products. Data were analyzed applying the ΔΔCt method and expressed as fold change vs. control.

### 2.4. Enzyme-Linked Immunosorbent Assay (ELISA)

Levels of BDNF in medium from HMC3 cells plated in 96-well microplates were determined using the Biosensis^®^ BDNF RapidTM ELISA kit (Biosensis Pty Ltd., Thebarton, SA, Australia), strictly following the manufacturer’s instructions. Absorbance at 450 nm was measured with a VarioskanTM Flash Multimode Reader.

### 2.5. Western Blot

Cells were collected and lysed in M-PER^®^ Mammalian Protein Extraction Reagent (Thermofisher Scientific, Waltham, MA, USA) supplemented with anti-protease and anti-phosphatase cocktails (Sigma-Merck, Darmstadt, Germany). Samples were sonicated, and centrifuged at high speed for 5 min at 4 °C, and protein concentration was determined by a Bradford reagent (Sigma-Merck, Darmstadt, Germany), according to the manufacturer’s instructions. Absorbance was measured with a VarioskanTM Flash Multimode Reader. Nuclear and cytoplasmic fractions were extracted using the Subcellular Protein Fractionation Kit for Cultured Cells (Thermofisher Scientific, Waltham, MA, USA), according to the manufacturer’s instructions. Sodium dodecyl sulfate-poly-acrylamide gel electrophoresis (SDS-PAGE) was performed by loading equal amounts of protein extracts per experiment on pre-cast “any-kDa” or 4–20% gradient gels (Bio-Rad, Hercules, CA, USA) followed by transfer to nitrocellulose membrane (Hybond ECL, Amersham Biosciences Europe GmbH, Milan, Italy) using a Transblot semidry transfer cell (Bio-Rad, Hercules, CA, USA). Membranes were blocked with a Blocker FL Fluorescent Blocking buffer (Thermofisher Scientific, Waltham, MA, USA) and incubated with primary antibodies overnight at 4 °C. The primary antibodies used were mouse anti-BDNF (1:900; Thermofisher Scientific, Waltham, MA, USA, Cat. No. MA5-34960), rabbit anti-SIRT1(H300) (1:400; Santa Cruz Biotechnologies, Santa Cruz, CA, USA, Cat. No. sc-15404), rabbit anti-NF-kBp65 (1:400; Thermofisher Scientific, Waltham, MA, USA, Cat. No. PA1-186), rabbit anti-β-actin (1:5000; Sigma-Merck, Darmstadt, Germany, Cat. No. A2066), mouse anti-glyceraldehyde 3-phosphate dehydrogenase (GAPDH) (1:5000; Millipore, Billerica, MA, USA, Cat. No. MAB374), and mouse anti-lamin B1 (1:1000; Santa Cruz Biotechnologies, Santa Cruz, CA, USA, Cat. No. sc-365214). Membranes were then washed and exposed to appropriate secondary antibodies for 45 min at RT as follows: AlexaFluor (AF) 647-conjugated anti-rabbit (1:2000; Thermofisher Scientific, Waltham, MA, USA), AF488 Plus-conjugated anti-rabbit (1:2000; Thermofisher Scientific, Waltham, MA, USA), and AF488 Plus-conjugated anti-mouse (1:5000; Thermofisher Scientific, Waltham, MA, USA). The detection of specific bands was carried out using the iBright FL1500 Imaging System (Thermofisher Scientific, Waltham, MA, USA). Band intensity was analyzed using the ImageJ software, developed by the National Institutes of Health (NIH) and in the public domain.

### 2.6. Immunoprecipitation (IP) & SIRT1 Activity Assay

Cell lysates were obtained as described in the Western blot section above. An amount of 350 µg of extracted proteins was diluted in a final volume of 500 µL with M-PER lysis buffer and incubated with 2 µg of rabbit anti-SIRT1(H300) primary antibody (1:400; Santa Cruz Biotechnologies, Santa Cruz, CA, USA, Cat. No. sc-15404) for 24 h at 4 °C. Next, 20 µL of Protein A/G PLUS-Agarose beads (Santa Cruz Biotechnologies, Santa Cruz, CA, USA, Sc-2002) were added, followed by incubation at 4 °C overnight. The mixture was centrifuged at 2500 rpm for 5 min at 4 °C. The supernatant was discarded, and the co-IP products were washed five times with PBS. After the final wash, the precipitates were resuspended in 30 μL of assay buffer from the SIRT1 activity assay kit. Enzyme activity was assayed with SIRT1 Fluorometric Drug Discovery Kit (Enzo Life Sciences Inc., Farmingdale, NY, USA) according to the manufacturer’s instructions.

### 2.7. Immunocytochemistry

Cells were fixed using InsideFix Solution (Miltenyi Biotec, Bologna, Italy) and incubated overnight at 4 °C with primary antibodies diluted in InsidePerm solution (Miltenyi, Bologna, Italy). The antibodies used were rabbit anti-SIRT1(H300) (1:400; Santa Cruz Biotechnologies, Santa Cruz, CA, USA, Cat. No. sc-15404) and rabbit anti-acetyl-NF-kB p65 (Lys310) (1:30; Cell Signaling, Danvers, MA, USA, Cat. No. 3045). After washing, cells were incubated with secondary antibodies, diluted in InsidePerm solution, for 45 min RT. The secondary antibodies used were AF488-anti-mouse (1:300; Thermofisher Scientific, Waltham, MA, USA) and AF488-anti-rabbit (1:300; Thermofisher Scientific, Waltham, MA, USA). After washing, slides were mounted with 4′,6-diamidino-2-phenylindole (DAPI)-containing mounting solution (Sigma-Merck, Darmstadt, Germany). Digital images were captured with a Zeiss Observer.Z1 microscope equipped with the Apotome.2 acquisition system (Zeiss, Oberkochen, Germany). The number of immunopositive cells with nuclear SIRT1 was determined by cell counting in at least five randomly selected fields/well.

### 2.8. Statistical Analysis

All data were from three or more independent experiments run at least in triplicate. All experimental values are presented as the mean ± SEM. Statistical analyses were performed, as appropriate, by Student’s t-test and one- or two-way ANOVA followed by Newman–Keuls post-hoc test using GraphPad Prism Software (GraphPad Software, San Diego, CA, USA). *p* < 0.05 was the criterion for statistical significance.

## 3. Results

### 3.1. Microglia Respond to Aβ42 with Transient SIRT1-Mediated BDNF Upregulation That Is Prolonged by Melatonin

The ability of HMC3 microglia to upregulate BDNF in response to a short exposure to Aβ42 was initially analyzed at the mRNA level by qRT-PCR. To this end, a low concentration of 0.2 μM and a higher one of 2 μM were initially tested. Results confirmed that only the lowest concentration (0.2 μM) induced a significant short-term increase of BDNF mRNA at 6 h (fold change of 1.42 ± 0.06 vs. C). In contrast, at the higher concentration of 2 μM, this effect was not present (fold change of 0.96 ± 0.12 vs. C). Based on this preliminary evidence, subsequent experiments were carried out using 0.2 μM of Aβ42.

**Figure 1:**
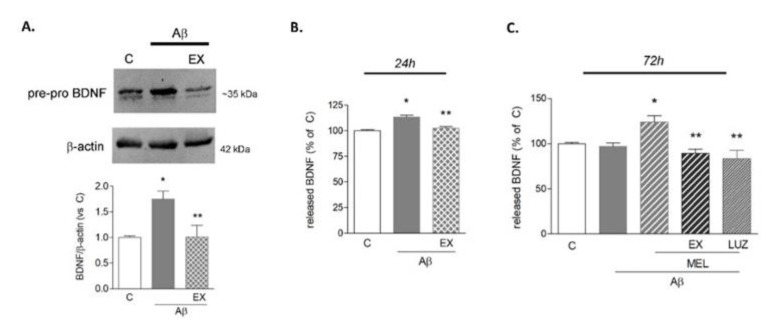
BDNF expression in HMC3 cells upon Aβ42 exposure. In (**A**,**B**), cells were treated with Aβ42 (0.2 μM) alone or in combination with SIRT1-selective inhibitor EX527 (EX, 5 μM). In (**A**), Western blot analysis of the intracellular content of BDNF at 6 h in the presence of brefeldin A (5 μg/mL). A representative blot is shown. ELISA determinations of released BDNF are reported at 24 h (**B**) and 72 h (**C**). In panel (**C**), melatonin (MEL, 1 μM) was added to Aβ42, alone or in combination with EX or luzindole (LUZ, 25 μM). Results are the mean ± SEM of 3–5 independent experiments. * *p* < 0.05 vs. C and ** *p* < 0.05 vs. Aβ (**B**) or vs. Aβ+MEL (**C**) by one-way ANOVA followed by Newman Keuls test for significance.

Western blot analysis was then performed to determine protein levels of BDNF shortly after Aβ42 exposure. In these experiments, brefeldin A (5 μg/mL) was added during the last 3 h of treatment, in order to prevent BDNF release and maximize its detection. Because of brefeldin A interference with the protein maturation pathway, a pre-pro isoform of BDNF of about 35 kDa was detected. Microglia responded to Aβ42 with a significant increase in BDNF protein expression (Figure 1A). To examine the involvement of SIRT1 as a mediator of this effect, selective SIRT1 inhibitor EX527 (5 μM) was added in combination with Aβ42. Results show that in these conditions, the BDNF increase was prevented (Figure 1A). Released BDNF levels were then assayed by ELISA in a conditioned medium at 6 and 24 h and after a prolonged exposure to Aβ42 for 72 h. While no effect was detected at 6 h (not shown), released BDNF levels were significantly augmented compared to control at 24 h, an effect sensitive to EX527 (5 μM; Figure 1B). When treatments were prolonged to 72 h, microglia lost their ability to upregulate BDNF release in response to Aβ42 (Figure 1C). Melatonin was thus tested in these conditions for its ability to contrast BDNF reduction. As shown in Figure 1C, in the presence of 1 μM of melatonin, BDNF levels were still significantly higher than in control or Aβ42-treated cells. Notably, melatonin’s effect was prevented by the addition of EX527 (5 μM) and of the mixed MT1/MT2 melatonin receptor antagonist luzindole (25 μM), indicating a SIRT1-mediated and receptor-dependent action.

### 3.2. Microglia Undergo a Pro-Inflammatory Switch Following Prolonged Aβ42 Exposure

**Figure 2:**
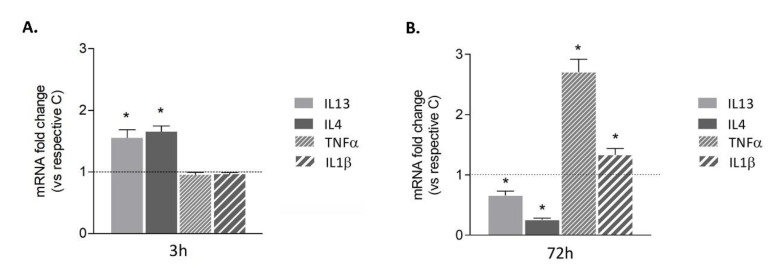
Time course of HMC3 microglial polarization. Cells were treated with 0.2 μM of Aβ42. 3 h (**A**) or 72 h (**B**). Expression of anti-inflammatory (IL4 and IL13) and pro-inflammatory (TNFα and IL1β) markers was investigated by qRT-PCR. Dotted lines indicate control values. Results are the mean ± SEM of three independent experiments. * *p* < 0.05 vs. respective control by Student’s *t*-test for significance.

To correlate transient BDNF induction after exposure to Aβ42 with the state of polarization of human HMC3 microglial cells, gene expression of anti- and pro-inflammatory markers was evaluated by qRT-PCR at 3 and 72 h. The anti-inflammatory markers interleukin (IL) 13 and IL4 were significantly induced shortly after exposure to Aβ42 (Figure 2A), but were downregulated after prolonged treatment (Figure 2B). On the contrary, pro-inflammatory markers TNFα and IL1β were not modified after short exposure to Aβ42 (Figure 2A), but were increased after prolonged exposure (Figure 2B). This is indicative of a microglial switch towards a pro-inflammatory phenotype after prolonged Aβ42 exposure.

### 3.3. Melatonin Prolongs Transient Aβ42-Induced Upregulation of SIRT1 Activity and Expression

**Figure 3:**
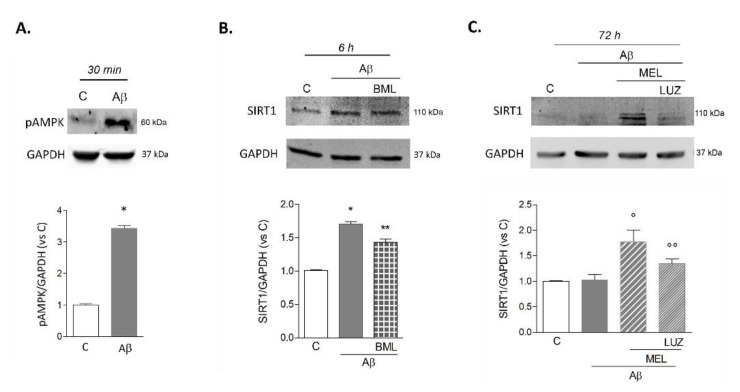
Involvement of pAMPK and time-course of SIRT1 expression in HMC3 cells upon Aβ42 exposure. Intracellular content of pAMPK (**A**) and SIRT1 (**B**,**C**) was evaluated by Western blot analysis at the time points indicated. Cells were exposed to either Aβ42 (0.2 μM) alone or in combination with AMPK inhibitor BML-275 (BML, 2 μM; **B**), with melatonin (MEL, 1 µM; **C**) or with MEL+luzindole (LUZ, 25 µM; **C**). Representative blots are shown. Results are the mean ± SEM of 3–5 independent experiments. * *p* < 0.05 vs. C by Student’s t-test (**A**), * *p* < 0.05 vs. C, ** *p* < 0.05 vs. Aβ (**B**); ° *p* < 0.05 vs. C and Aβ, °° *p* < 0.05 vs. Aβ+MEL (**C**) by one-way ANOVA followed by Newman Keuls test for significance.

Given the involvement of SIRT1 in mediating Aβ42- and melatonin-induced effects on BDNF levels, the time course of its expression was characterized in more detail. Based on the well-established interdependence of SIRT1 with the activation of the AMP-regulated protein kinase (AMPK) pathway, we first analyzed phosphorylated AMPK (pAMPK) induction by Western blot. Thirty minutes after exposure to Aβ42, pAMPK was significantly upregulated (Figure 3A). Next, we examined the modulation of SIRT1 levels in response to Aβ42 and the effects of pharmacological AMPK blockade with BML-275. Western blot showed that within 6 h, SIRT1 content was increased, an effect slightly but significantly reduced by BML-275 (2 μM; Figure 3B). After 72 h, SIRT1 returned to control levels in microglia exposed to Aβ42 alone (Figure 3C). Again, we tested the effects of melatonin (1 μM) in combination with Aβ42. As shown in Figure 3C, in these conditions SIRT1 levels remained significantly higher than control or Aβ42-treated cells. This effect was sensitive to MT receptors antagonist luzindole (25 μM; Figure 3C).

**Figure 4:**
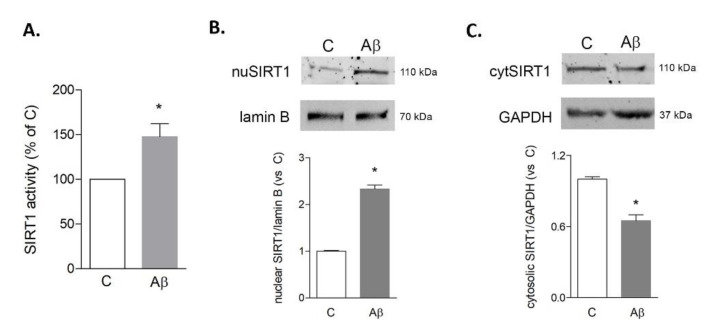
Activity and subcellular localization of SIRT1 in HMC3 cells upon Aβ42 exposure. Cells were treated for 6 h with Aβ42 (0.2 µM). SIRT1 enzymatic activity was evaluated in lysates immunoprecipitated for SIRT1 (**A**). Nuclear (**B**) and cytosolic (**C**) expression of SIRT1 were investigated by Western blot analysis on purified fractions. Representative blots are shown. Results are the mean ± SEM of three independent experiments. * *p* < 0.05 vs. C by Student’s *t*-test for significance.

In order to analyze the activation of SIRT1, we carried out an enzymatic activity assay and Western analysis of its nuclear localization. The activity assay was selectively performed on SIRT1 immunoprecipitates in order to exclude contribution from other sirtuins. Results confirmed that after 6 h of exposure to Aβ42 (0.2 μM), SIRT1 activity was significantly increased compared to control (Figure 4A). In agreement, analysis of the subcellular localization of upregulated SIRT1 showed an increase in the nuclear fraction (Figure 4B) and a parallel reduction in the cytosolic fraction (Figure 4C).

**Figure 5:**
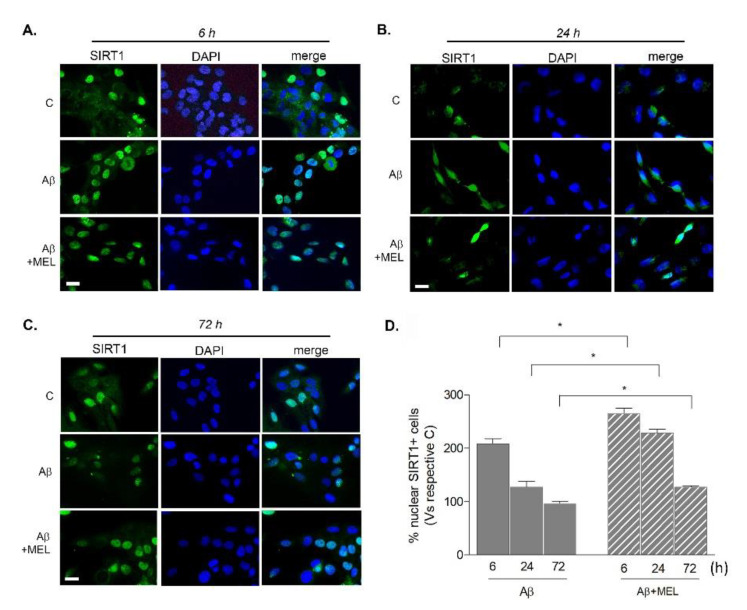
Time course of SIRT1 nuclear localization in HMC3 cells upon Aβ42 exposure. Cells were treated with 0.2 µM of Aβ42 alone or in combination with 1 µM melatonin (MEL) for 6, 24, or 72 h. In panels (**A**–**C**), representative images of immunostaining for SIRT1 (green) and nuclear counterstaining with DAPI (blue). Scale bar = 40 μm. In panel (**D**), graph reporting the percentage of nuclear SIRT1-positive cells over total SIRT1-positive cells, each vs. respective control, set as 100%. Results are the mean ± SEM of 3–5 independent experiments. * *p* < 0.05 vs. treatment with Aβ alone at corresponding time points (two-way ANOVA followed by Newman–Keuls test for significance; treatment vs. time).

To further strengthen this result and monitor the sub-cellular localization of SIRT1 in time, cells were immunostained and counted for nuclear SIRT1 positivity. Representative images of SIRT1-labeled cells (green) counterstained with DAPI (blue) are reported in Figure 5A–C. After 6 h of exposure to Aβ42 alone, the population of nuclear SIRT1-positive cells was increased by 108% over the control (Figure 5D). When in combination with melatonin, Aβ induced a significantly more pronounced increase (165% over the control; Figure 5D). With Aβ42 alone, this effect was progressively reduced at 24 h (26.7% over the control; Figure 5D) and disappeared at 72 h (−5% vs. control; Figure 5D), but remained higher when in combination with melatonin (+128% vs. control at 24 h and +27% vs. control at 72 h; Figure 5D). Overall, these results confirm that SIRT1 is shortly but transiently upregulated by microglia in response to Aβ42 and that melatonin is able to potentiate and prolong this effect.

### 3.4. Melatonin Reduces Microglial NF-kB Expression Induced by Prolonged Aβ42 Exposure

**Figure 6:**
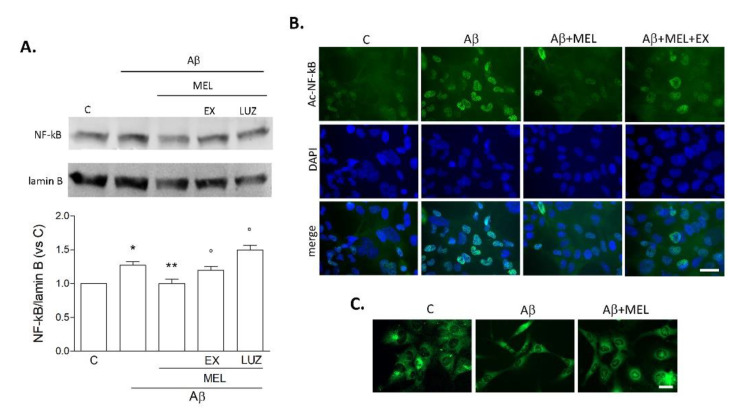
Pro-inflammatory switch of HMC3 cells upon prolonged Aβ42 exposure. Cells were treated for 72 h with Aβ42 alone (0.2 μM), in combination with melatonin (MEL, 1 μM), MEL+EX527 (EX; 5 μM), or MEL+luzindole (LUZ; 25 μM). In (**A**), Western blot of NF-kB p65 on nuclear fractions. Results are the mean ± SEM of three independent experiments, and a representative blot is shown. * *p* < 0.05 vs. C, ** *p* <0.05 vs. Aβ, ° *p* < 0.05 vs. Aβ+MEL by one-way ANOVA followed by Newman–Keuls test for significance. In (**B**), representative images of immunostaining for acetyl-Lys310-NF-kB p65 (green) with DAPI counterstaining (blue; scale bar = 40 μm). In (**C**), morphological appearance of cells stained with fluorescent dye FM 1–43 (5 μM for 15 min; scale bar = 50 μm).

We next focused on NF-kB, a well-known target of SIRT1, with a crucial role in microglial pro-inflammatory activation. Western blot analysis on nuclear fractions confirmed an increase of NF-kB p65 after a 72 h-exposure to Aβ42 (Figure 6A). Addition of melatonin (1 μM) prevented this effect in an EX527- (5 μM) and luzindole- (25 μM) dependent fashion (Figure 6A). Since SIRT1 can directly inactivate NF-kB by deacetylation at lysine 310 (Lys310), immunostaining of acetylated NF-kB p65 was performed (green; Figure 6B). Results confirmed an increase of nuclear acetylated NF-kB-positive cells following Aβ42 exposure for 72 h. This effect was counteracted by melatonin but reappeared when cells were exposed to Aβ42+melatonin under a blockade of SIRT1 by EX527 (Figure 6B). The long-term effects of Aβ42 were also accompanied by slight morphological changes, as visualized by staining with fluorescent dye FM 1–43 (5 μM for 15 min). As shown in Figure 6C, HMC3 cells exhibited an elongated, bipolar phenotype upon exposure to Aβ42 (0.2 µM), which was partially reversed by treatment with melatonin.

## 4. Discussion

Microglia are the resident immune cells in the brain and play a crucial role of surveillance against micro-environmental changes that could pose a threat to brain homeostasis. Microglial activation is finely balanced between pro- and anti-inflammatory phenotypes that act in concert to restore homeostasis through self-limited inflammatory events. However, this balance can be disrupted under chronic toxicity, leading to a switch from protective to detrimental [45,46,47,48]. This has been proposed to occur also in AD, where progressive accumulation of Aβ42 oligomers, over a time span of up to two decades, slowly but relentlessly leads to progressive cellular distress and chronic toxicity. This in time will push microglia towards an inflammation-sustaining phenotype [5,49,50,51].

The focus on microglial contribution in AD has been especially, though mainly unsuccessfully, aimed at contrasting inflammation [52,53]. However, targeting microglia to enhance their initial protective features, rather than entirely turning off their activation, appears as an appealing strategy. To this end, the very initial responses of microglia to Aβ still need to be fully characterized.

Our present study moves from our previous work, where we established in vitro models of slow-developing neuronal damage using low concentrations of oligomeric Aβ [11,54]. This allowed us to show that early Aβ-induced microglial BDNF was the mediator of an early compensatory and protective response against Aβ toxicity in neuronal cells [11,54]. On these bases, the next step was to study the time course and the mechanisms underlying Aβ-induced BDNF increase in microglia. For our purposes, we were now able to use microglia of human origin, the HMC3 cell line [55]. This appears relevant due to the different responses between murine and human microglia, as recently pointed out [56]. Notably, the early increase in BDNF and the time-dependent fluctuations in anti- and pro-inflammatory gene expression confirmed that our model, based on low Aβ42 as a light noxious stimulus, well recapitulated the dual microglial activation and the intrinsic decline of the initial neurotrophic response. In agreement, BDNF reduction has been largely linked to cognitive decline in AD patients [57,58,59] and preclinical in vivo models, where its administration proved to be neuro- and synapto-protective [60,61,62]. In vitro models provided concordant observations [63,64,65]. Notably, it was also shown that aging itself can cause a decline in microglial BDNF, which correlates with a pro-inflammatory switch [66].

Since the ability to support BDNF-producing, protective microglia entails the identification of an appropriate target, we here contemplated a role for SIRT1. In order to fully characterize the involvement of SIRT1 in our model and to exclude the contribution of other cellular sirtuins to the measured deacetylase activity, an in vitro activity assay was firstly carried out using SIRT1-immunoprecipitated lysates. Furthermore, SIRT1 nuclear localization was evaluated as an index of enzyme activation. Evidence from different cell types shows in fact that SIRT1 can shuttle between the nucleus and cytoplasm, exerting differential functions [67,68]. It has been pointed out that SIRT1 activity may be hampered depending on local NAD^+^ availability [69]. However, this was not the case in our conditions, as shown by the inhibitory effects of EX527. Finally, because SIRT1 activity has been reported to be interconnected with the activation of the AMPK pathway [70,71], we confirmed AMPK involvement both by looking at its direct induction by Aβ42, and by evidencing the effects of its pharmacological blockade on SIRT1 expression. The choice to focus on SIRT1 was based on its established neuroprotective role, particularly relevant against aging and age-related diseases. SIRT1 has multiple beneficial actions in the central nervous system [72], including modulation of synaptic plasticity, learning, and memory [73,74], anti-apoptotic activity, and antioxidant and anti-inflammatory properties [75]. Deacetylation of key transcription factors such as forkhead box O3 (FOXO3), peroxisome proliferator-activated receptor γ (PPARγ), and NF-kB appear mainly involved in these effects [39,75,76,77]. Also in AD animal models, activation or overexpression of SIRT1 was linked to neuroprotection and improved cognitive function [78,79,80], whereas cognitive deficits in SIRT1 knockout mice were aggravated (Bonda et al., 2011). Interestingly, in AD patients, levels of SIRT1 appeared reduced in the serum [81], hippocampus [82], and cortex [24] and inversely correlated with neuropathological changes [23]. These data are supported by in vitro studies showing that SIRT1 directly affected Aβ production in neurons [83], promoted Aβ clearance in astrocytes [84], and inhibited inflammatory signaling in microglia [22].

Our results showed that in microglia, SIRT1 peaked early, but transiently, after Aβ42 exposure, mediating an initial BDNF-sustained neurotrophic response. In an attempt to prolong the beneficial microglial polarization, we considered as a potential candidate melatonin, a safe molecule that easily crosses the blood–brain barrier [31,85]. Indeed, melatonin in combination with Aβ prolonged the BDNF-producing state of human microglial cells, an effect majorly dependent on the induction of SIRT1 and on surface signaling through MT1/MT2 receptors. At the same time, melatonin prevented nuclear induction of pro-inflammatory transcription factor NF-kB and, importantly, attenuated its acetylation at Lys310. This is consistent with the reported ability of SIRT1 to inactivate NF-kB by the removal of the acetylic group in Lys 310 [86], which is required for NF-kB full transcriptional activity on target promoters [87]. Indeed, NF-kB inactivation prevents the microglia pro-inflammatory switch and appears relevant for neuroprotection in AD, as previously shown [21,22,88]. In our hands, the Aβ-induced microglial switch correlated with a slight trend towards a more elongated cell morphology, which melatonin was able to prevent. Data on morphological changes connected to pro-inflammatory activation of the HMC3 cell line are currently scarce and somewhat discordant. In one study, HMC3 cells appeared elongated and bipolar following stimulation with IFNγ+IL1β for 24 h [89]. In another report, activation with a high concentration of Aβ42 (5 µM) for 24 h corresponded to the acquisition of an amoeboid shape [90].

Overall, our results on melatonin’s effects are in agreement with its reported multiple beneficial actions that go well beyond a mere regulation of circadian rhythms. The compound is in fact endowed with anti-inflammatory, antioxidant, and neuroprotective activity against a number of neurodegenerative conditions that share neuroinflammatory features [35,91], including Parkinson’s disease [92], hypoxia [29,93], amyotrophic lateral sclerosis [94], traumatic brain injury, spinal cord injury [95], and neuropsychiatric disorders [96]. A role for melatonin has convincingly emerged also in AD, where an inverse correlation between melatonin levels and disease progression has been reported in patients, along with sleep–wake cycle disturbances [43,97]. This could be indicative of a potential loss of endogenous protection when melatonin levels are reduced. Preclinical studies on AD transgenic mice models confirmed the rescue of cognitive functions by melatonin administration, also in association with AD-approved symptomatic drug memantine [98,99,100,101]. However, molecular mechanisms involved in melatonin-mediated neuroprotection have been majorly investigated in neurons, whereas studies on glial cells are limited. Melatonin was reported to suppress the hippocampal glial activation induced by Aβ25-35 in rats [102], but, to our knowledge, there are no other detailed studies on glial cells as potential targets for melatonin in AD. We here showed for the first time that the addition of melatonin to Aβ was efficient in prolonging the peak in SIRT1 and related BDNF expression, maintaining human microglia in an anti-inflammatory state.

## 5. Conclusions

**Figure 7:**
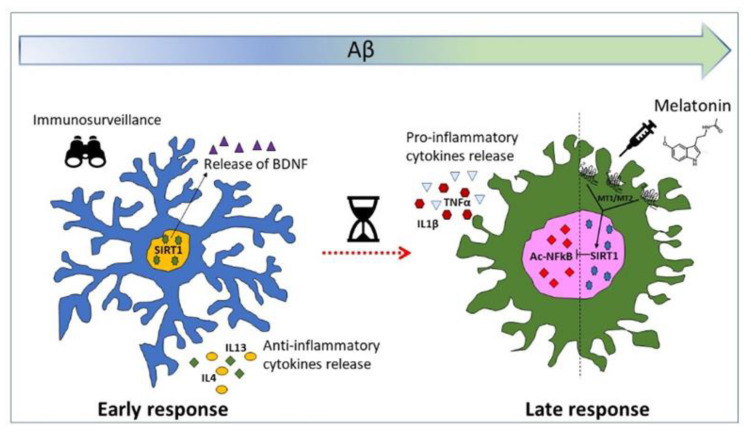
Dual response of microglia to Aβ challenge.

Long before AD patients enter the clinical phase, attempted protective responses take place at the cellular level that may be important in determining some degree of resilience to neurodegeneration [103,104]. Among these, microglial protective activation seems to play a key role. In the present study, we demonstrated that following a subtle challenge with Aβ, human microglial cells upregulate BDNF synthesis and release, via induction of deacetylase SIRT1. This effect is accompanied by anti-inflammatory features, but is only transient. We here show that the addition of melatonin can maintain high SIRT1/BDNF levels in the presence of Aβ for a prolonged time (Figure 7). Our study thus identifies microglial SIRT1 as a potential target in AD and highlights a therapeutic potential for melatonin as a SIRT1/BDNF inducer in microglial cells.

## Figures and Tables

**Table 1:** Primers used for qRT-PCR.

Gene	Primer	Cat. No.
BDNF	Hs_BDNF_1_SG QuantiTect Primer Assay	QT00235368
IL-13	Hs_IL13_1_SG QuantiTect Primer Assay	QT00000511
IL-4	Hs_IL4_1_SG QuantiTect Primer Assay	QT00012565
TNFα	Hs_TNF_1_SG QuantiTect Primer Assay	QT00029162
IL-1β	Hs_IL1B_1_SG QuantiTect Primer Assay	QT00021385
RPLP0	Hs_RPLP0_1_SG QuantiTect Primer Assay	QT00075012
